# Guanidinium bromide–18-crown-6 (2/1)

**DOI:** 10.1107/S1600536812017394

**Published:** 2012-04-25

**Authors:** Yu-feng Wang

**Affiliations:** a Ordered Matter Science Research Center, Southeast University, Nanjing 211189, People’s Republic of China

## Abstract

In the title compound, 2CH_6_N_3_^+^·2Br^−^·C_12_H_24_O_6_, the 18-crown-6 mol­ecule lies about an inversion center, whereas the guanidinium cation and bromide anion are in general positions. The guanidinium cations link with the bromide anions and the crown ether mol­ecules *via* N—H⋯O and N—H⋯Br hydrogen bonds, thus forming a three-dimensional network.

## Related literature

For applications of crown ethers, see: Clark *et al.* (1998 ▶). For ferroelectric metal-organic compounds, see: Fu *et al.* (2009 ▶, 2011 ▶); Ye *et al.* (2006 ▶); Zhang *et al.* (2008 ▶, 2010 ▶). For structures of 18-crown-6 clathrates, see: Zhang & Zhao (2011 ▶); Ge & Zhao (2010 ▶)
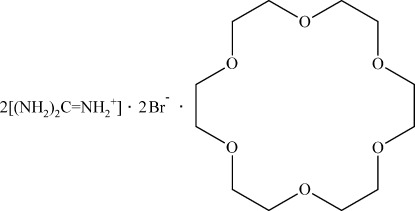


## Experimental

### 

#### Crystal data

2CH_6_N_3_^+^·2Br^−^·C_12_H_24_O_6_
*M* *_r_* = 544.31
Monoclinic, 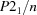

*a* = 8.9354 (18) Å
*b* = 9.860 (2) Å
*c* = 14.306 (3) Å
β = 101.39 (3)°
*V* = 1235.6 (4) Å^3^
*Z* = 2
Mo *K*α radiation
μ = 3.32 mm^−1^
*T* = 293 K
0.20 × 0.20 × 0.20 mm

#### Data collection

Rigaku SCXmini diffractometer
Absorption correction: multi-scan (*CrystalClear*; Rigaku, 2005 ▶) *T*_min_ = 0.936, *T*_max_ = 0.937
12464 measured reflections
2835 independent reflections
1968 reflections with *I* > 2σ(*I*)
*R*_int_ = 0.077

#### Refinement

*R*[*F* ^2^ > 2σ(*F* ^2^)] = 0.053
*wR*(*F* ^2^) = 0.127
*S* = 1.11
2835 reflections
127 parameters
H-atom parameters constrained
Δρ_max_ = 0.36 e Å^−3^
Δρ_min_ = −0.79 e Å^−3^

### 

Data collection: *CrystalClear* (Rigaku, 2005 ▶); cell refinement: *CrystalClear*; data reduction: *CrystalClear*; program(s) used to solve structure: *SHELXTL* (Sheldrick, 2008 ▶); program(s) used to refine structure: *SHELXTL*; molecular graphics: *SHELXTL*; software used to prepare material for publication: *SHELXTL*.

## Supplementary Material

Crystal structure: contains datablock(s) I, global. DOI: 10.1107/S1600536812017394/yk2049sup1.cif

Structure factors: contains datablock(s) I. DOI: 10.1107/S1600536812017394/yk2049Isup2.hkl

Additional supplementary materials:  crystallographic information; 3D view; checkCIF report

## Figures and Tables

**Table 1:** Hydrogen-bond geometry (Å, °)

*D*—H⋯*A*	*D*—H	H⋯*A*	*D*⋯*A*	*D*—H⋯*A*
N1—H1*A*⋯Br1^i^	0.86	2.68	3.470 (3)	154
N1—H1*B*⋯O2	0.86	2.14	2.938 (4)	155
N2—H2*C*⋯O2	0.86	2.40	3.127 (5)	143
N2—H2*D*⋯Br1	0.86	2.82	3.582 (4)	149
N3—H3*D*⋯Br1	0.86	2.53	3.354 (3)	162
N3—H3*C*⋯Br1^i^	0.86	2.64	3.440 (3)	156

Symmetry code: (i) 
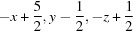
.

**Fig. 1.**
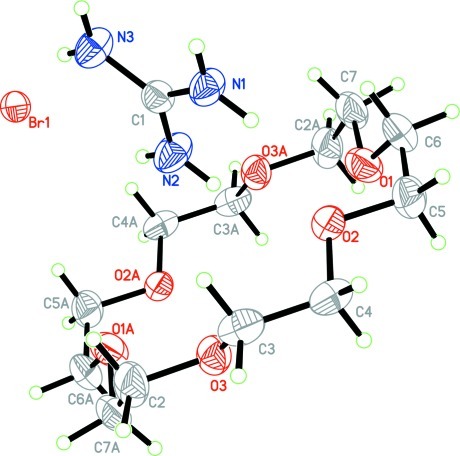
The asymmetric unit of the title compound, showing the atomic numbering scheme. Displacement ellipsoids are drawn at the 30% probability level.

**Fig. 2.**
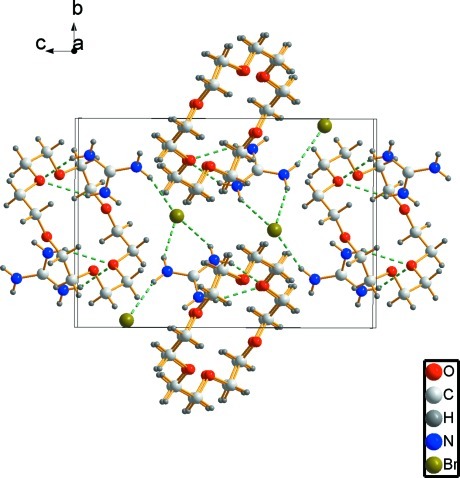
A view of the packing of the title compound viewed along the a axis. Dashed lines indicate hydrogen bonds.

## supplementary crystallographic information

### Comment

Recent years, crown ethers have attracted much attention because of their wide application in catalysis, solvent extraction, separation of isotopes, host–guest and supramolecular chemistry (Clark *et al.*, 1998). Several 18-crown-6 clathrates were discovered to be dielectric-ferroelectric materials (Fu *et al.*, 2011), hence we designed the title compound in attempts to find new hydrogen-bonded dielectric materials. Dielectric-ferroelectric materials, comprising organic ligands, metal-organic coordination compounds and organic-inorganic hybrids almost show temperature dependence of their dielectric constants (Fu *et al.*, 2009; Zhang *et al.*, 2010; Zhang *et al.*, 2008; Ye *et al.*, 2006). Unfortunately, the study of temperature dependence of dielectric constant of the title compound indicates that the permittivity is basically temperature-independent below its melting point (395K—396K). Herein we descibe the crystal structure of this compound.

At room temperature (25°C), the single-crystal X-ray diffraction reveals that the asymmetric unit of the title compound consists of a guanidinium cation, a bromide anion and a half of 18-crown-6 molecule (Fig. 1). The three NH_2_-groups of guanidinium interact with the oxygen atoms of crown ether molecule and with two bromide anions through two N—H···O and N—H···Br hydrogen bonds (Table 1), thus forming a three-dimensional network (Fig. 2).

### Experimental

The hydrobromic acid (0.81 g, 10 mmol) and guanidinium carbonate (0.9 g, 5 mmol) were dissolved in 30 ml of water and the solution was combined with methanol solution of dibenzo-18-crown-6 (3.6 g 10 mmol). The mixture was stirred for 30 min to complete the reaction, and good quality blocky single crystals were obtained by slow evaporation of the filtrate after two weeks (the chemical yield 72%).

### Refinement

Amino H atoms were located in a difference Fourier map and refined isotropically. Other H atoms were placed in geometrically idealized positions and constrained to ride on their parent atoms with C—H = 0.97 Å and N—H = 0.86 Å, *U*_iso_(H) = 1.2*U*_iso_(C,N).

### Crystal data

**Table:**

2CH_6_N_3_^+^·2Br^−^·C_12_H_24_O_6_	*F*(000) = 560
*M**_r_* = 544.31	*D*_x_ = 1.463 Mg m^−^^3^
Monoclinic, *P*2_1_/*n*	Mo *K*α radiation, λ = 0.71073 Å
Hall symbol: -P 2yn	Cell parameters from 3638 reflections
*a* = 8.9354 (18) Å	θ = 3.0–27.5°
*b* = 9.860 (2) Å	µ = 3.32 mm^−^^1^
*c* = 14.306 (3) Å	*T* = 293 K
β = 101.39 (3)°	Block, colourless
*V* = 1235.6 (4) Å^3^	0.20 × 0.20 × 0.20 mm
*Z* = 2	

### Data collection

**Table:**

Rigaku SCXmini diffractometer	2835 independent reflections
Radiation source: fine-focus sealed tube	1968 reflections with *I* > 2σ(*I*)
Graphite monochromator	*R*_int_ = 0.077
ω scans	θ_max_ = 27.5°, θ_min_ = 3.1°
Absorption correction: multi-scan (*CrystalClear*; Rigaku, 2005)	*h* = −11→11
*T*_min_ = 0.936, *T*_max_ = 0.937	*k* = −12→12
12464 measured reflections	*l* = −18→18

### Refinement

**Table:**

Refinement on *F*^2^	Primary atom site location: structure-invariant direct methods
Least-squares matrix: full	Secondary atom site location: difference Fourier map
*R*[*F*^2^ > 2σ(*F*^2^)] = 0.053	Hydrogen site location: inferred from neighbouring sites
*wR*(*F*^2^) = 0.127	H-atom parameters constrained
*S* = 1.11	*w* = 1/[σ^2^(*F*_o_^2^) + (0.0528*P*)^2^] where *P* = (*F*_o_^2^ + 2*F*_c_^2^)/3
2835 reflections	(Δ/σ)_max_ = 0.001
127 parameters	Δρ_max_ = 0.36 e Å^−^^3^
0 restraints	Δρ_min_ = −0.79 e Å^−^^3^

### Special details

**Table:**

Geometry. All s.u.'s (except the s.u. in the dihedral angle between two l.s. planes) are estimated using the full covariance matrix. The cell s.u.'s are taken into account individually in the estimation of s.u.'s in distances, angles and torsion angles; correlations between s.u.'s in cell parameters are only used when they are defined by crystal symmetry. An approximate (isotropic) treatment of cell s.u.'s is used for estimating s.u.'s involving l.s. planes.
Refinement. Refinement of *F*^2^ against ALL reflections. The weighted *R*-factor *wR* and goodness of fit *S* are based on *F*^2^, conventional *R*-factors *R* are based on *F*, with *F* set to zero for negative *F*^2^. The threshold expression of *F*^2^ > σ(*F*^2^) is used only for calculating *R*-factors(gt) *etc*. and is not relevant to the choice of reflections for refinement. *R*-factors based on *F*^2^ are statistically about twice as large as those based on *F*, and *R*- factors based on ALL data will be even larger.

### Fractional atomic coordinates and isotropic or equivalent isotropic displacement parameters (Å^2^)

**Table:**

	*x*	*y*	*z*	*U*_iso_*/*U*_eq_	
O2	1.1811 (3)	−0.2005 (3)	0.62582 (18)	0.0572 (7)	
O3	1.2804 (3)	0.0677 (3)	0.6025 (2)	0.0649 (8)	
O1	0.8672 (3)	−0.2293 (3)	0.5560 (2)	0.0759 (9)	
C3	1.3789 (5)	−0.0401 (5)	0.6413 (4)	0.0701 (13)	
H3A	1.4677	−0.0043	0.6844	0.084*	
H3B	1.4135	−0.0887	0.5905	0.084*	
C6	0.9606 (6)	−0.3369 (5)	0.5989 (4)	0.0805 (14)	
H6A	1.0024	−0.3854	0.5509	0.097*	
H6B	0.9004	−0.4000	0.6282	0.097*	
C5	1.0855 (6)	−0.2815 (5)	0.6716 (3)	0.0748 (13)	
H5A	1.0439	−0.2269	0.7168	0.090*	
H5B	1.1441	−0.3549	0.7061	0.090*	
C4	1.2942 (5)	−0.1325 (4)	0.6931 (3)	0.0641 (12)	
H4A	1.3635	−0.1980	0.7292	0.077*	
H4B	1.2465	−0.0814	0.7372	0.077*	
C7	0.7498 (6)	−0.2745 (5)	0.4820 (4)	0.0882 (16)	
H7A	0.6934	−0.3471	0.5050	0.106*	
H7B	0.7932	−0.3093	0.4298	0.106*	
C2	1.3526 (5)	0.1611 (6)	0.5511 (4)	0.0848 (15)	
H2A	1.3809	0.1160	0.4969	0.102*	
H2B	1.4450	0.1949	0.5917	0.102*	
N2	1.1162 (4)	−0.1452 (4)	0.4063 (3)	0.0766 (11)	
H2C	1.1030	−0.1300	0.4633	0.092*	
H2D	1.0709	−0.0954	0.3599	0.092*	
Br1	1.01901 (4)	−0.03281 (4)	0.16380 (3)	0.05407 (18)	
N3	1.2257 (4)	−0.2677 (4)	0.3022 (2)	0.0643 (9)	
H3C	1.2841	−0.3325	0.2911	0.077*	
H3D	1.1801	−0.2174	0.2562	0.077*	
C1	1.2061 (4)	−0.2453 (4)	0.3900 (3)	0.0508 (9)	
N1	1.2761 (4)	−0.3225 (3)	0.4595 (2)	0.0592 (9)	
H1A	1.3344	−0.3871	0.4478	0.071*	
H1B	1.2638	−0.3085	0.5168	0.071*	

### Atomic displacement parameters (Å^2^)

**Table:**

	*U*^11^	*U*^22^	*U*^33^	*U*^12^	*U*^13^	*U*^23^
O2	0.0681 (18)	0.0558 (17)	0.0495 (15)	0.0000 (13)	0.0161 (13)	−0.0003 (13)
O3	0.0545 (17)	0.078 (2)	0.0651 (19)	−0.0106 (15)	0.0176 (14)	0.0009 (16)
O1	0.082 (2)	0.0625 (19)	0.079 (2)	−0.0205 (16)	0.0071 (17)	0.0001 (17)
C3	0.050 (3)	0.087 (3)	0.073 (3)	0.000 (2)	0.010 (2)	−0.027 (3)
C6	0.100 (4)	0.055 (3)	0.091 (4)	−0.019 (3)	0.032 (3)	0.009 (3)
C5	0.095 (3)	0.065 (3)	0.065 (3)	0.005 (3)	0.018 (3)	0.021 (2)
C4	0.071 (3)	0.067 (3)	0.050 (2)	0.017 (2)	0.002 (2)	−0.010 (2)
C7	0.106 (4)	0.087 (4)	0.068 (3)	−0.055 (3)	0.009 (3)	0.002 (3)
C2	0.060 (3)	0.122 (4)	0.074 (3)	−0.040 (3)	0.015 (2)	0.003 (3)
N2	0.101 (3)	0.074 (2)	0.059 (2)	0.034 (2)	0.026 (2)	0.0060 (19)
Br1	0.0649 (3)	0.0498 (3)	0.0474 (3)	0.00012 (18)	0.01084 (19)	0.00461 (18)
N3	0.070 (2)	0.076 (2)	0.045 (2)	0.0250 (18)	0.0076 (16)	0.0070 (17)
C1	0.050 (2)	0.049 (2)	0.052 (2)	−0.0038 (17)	0.0091 (18)	0.000 (2)
N1	0.075 (2)	0.060 (2)	0.0410 (18)	0.0135 (17)	0.0093 (16)	0.0063 (17)

### Geometric parameters (Å, º)

**Table:**

O2—C4	1.418 (5)	C7—C2^i^	1.464 (7)
O2—C5	1.420 (5)	C7—H7A	0.9700
O3—C2	1.411 (5)	C7—H7B	0.9700
O3—C3	1.421 (5)	C2—C7^i^	1.464 (7)
O1—C7	1.407 (5)	C2—H2A	0.9700
O1—C6	1.413 (5)	C2—H2B	0.9700
C3—C4	1.474 (6)	N2—C1	1.322 (5)
C3—H3A	0.9700	N2—H2C	0.8600
C3—H3B	0.9700	N2—H2D	0.8600
C6—C5	1.472 (7)	N3—C1	1.320 (5)
C6—H6A	0.9700	N3—H3C	0.8600
C6—H6B	0.9700	N3—H3D	0.8600
C5—H5A	0.9700	C1—N1	1.309 (5)
C5—H5B	0.9700	N1—H1A	0.8600
C4—H4A	0.9700	N1—H1B	0.8600
C4—H4B	0.9700		
			
C4—O2—C5	111.5 (3)	H4A—C4—H4B	108.4
C2—O3—C3	112.3 (4)	O1—C7—C2^i^	109.1 (4)
C7—O1—C6	112.1 (4)	O1—C7—H7A	109.9
O3—C3—C4	108.6 (3)	C2^i^—C7—H7A	109.9
O3—C3—H3A	110.0	O1—C7—H7B	109.9
C4—C3—H3A	110.0	C2^i^—C7—H7B	109.9
O3—C3—H3B	110.0	H7A—C7—H7B	108.3
C4—C3—H3B	110.0	O3—C2—C7^i^	110.3 (4)
H3A—C3—H3B	108.4	O3—C2—H2A	109.6
O1—C6—C5	109.2 (4)	C7^i^—C2—H2A	109.6
O1—C6—H6A	109.8	O3—C2—H2B	109.6
C5—C6—H6A	109.8	C7^i^—C2—H2B	109.6
O1—C6—H6B	109.8	H2A—C2—H2B	108.1
C5—C6—H6B	109.8	C1—N2—H2C	120.0
H6A—C6—H6B	108.3	C1—N2—H2D	120.0
O2—C5—C6	108.9 (4)	H2C—N2—H2D	120.0
O2—C5—H5A	109.9	C1—N3—H3C	120.0
C6—C5—H5A	109.9	C1—N3—H3D	120.0
O2—C5—H5B	109.9	H3C—N3—H3D	120.0
C6—C5—H5B	109.9	N1—C1—N3	119.5 (4)
H5A—C5—H5B	108.3	N1—C1—N2	121.0 (4)
O2—C4—C3	108.6 (3)	N3—C1—N2	119.5 (4)
O2—C4—H4A	110.0	C1—N1—H1A	120.0
C3—C4—H4A	110.0	C1—N1—H1B	120.0
O2—C4—H4B	110.0	H1A—N1—H1B	120.0
C3—C4—H4B	110.0		

Symmetry code: (i) −*x*+2, −*y*, −*z*+1.

### Hydrogen-bond geometry (Å, º)

**Table:**

*D*—H···*A*	*D*—H	H···*A*	*D*···*A*	*D*—H···*A*
N1—H1*A*···Br1^ii^	0.86	2.68	3.470 (3)	154
N1—H1*B*···O2	0.86	2.14	2.938 (4)	155
N2—H2*C*···O2	0.86	2.40	3.127 (5)	143
N2—H2*D*···Br1	0.86	2.82	3.582 (4)	149
N3—H3*D*···Br1	0.86	2.53	3.354 (3)	162
N3—H3*C*···Br1^ii^	0.86	2.64	3.440 (3)	156

Symmetry code: (ii) −*x*+5/2, *y*−1/2, −*z*+1/2.
